# Long-term evolution of immunological and virological parameters in patients undergoing treatments for HIV-1 infection at the Day Hospital of Bobo-Dioulasso, Burkina Faso

**DOI:** 10.11604/pamj.2023.44.63.38091

**Published:** 2023-02-01

**Authors:** Jessica Julie Chantal Samba, Yacouba Sawadogo, Herman Karim Sombie, Hervé Kafando, Wendinmi Arnaud Marie Joseph Zougmore, Maxime Damolga, Cheick Ahmed Ouattara, Armel Poda, Abdoul-Salam Ouedraogo

**Affiliations:** 1 Emerging and Re-emerging Pathogens Laboratory, Sourô Sanou University Hospital Center, Bobo-Dioulasso, Burkina Faso,; 2 Bacteriology-Virology Department, Sourô Sanou University Hospital, Bobo-Dioulasso, Burkina Faso,; 3 Laboratory of Molecular Biology and Genetics (LABIOGENE), Joseph Ki Zerbo University, Ouagadougou, Burkina Faso,; 4 Pietro Annigoni Biomolecular Research Center (CERBA), Ouagadougou, Burkina Faso,; 5 Laboratory Department, Dedougou Regional Hospital, Avenue of Governor Binger Dedougou, Burkina Faso,; 6 Department of Information, Research, Epidemiology and Planning, Sourô Sanou University Hospital Center, Bobo-Dioulasso, Burkina Faso,; 7 Faculty of Medicine and Pharmacy, Institute of Health Sciences, Nazi Boni University, Bobo-Dioulasso, Burkina Faso,; 8 Infectious Diseases Department, Sourô Sanou University Hospital Center, Bobo-Dioulasso, Burkina Faso

**Keywords:** Immunological markers, virological markers, antiretroviral treatment, Burkina Faso

## Abstract

**Introduction:**

antiretroviral therapy enables the suppression of the plasma viral load and the restoration of immune responses. Therapeutic failures are still observed in patients living with HIV despite the considerable benefits of antiretroviral therapy. This study aimed to describe the long-term evolution of immunological and virological parameters in patients undergoing treatments for HIV-1 at the Day Hospital of Bobo-Dioulasso in Burkina Faso.

**Methods:**

a retrospective descriptive and analytical study covering 10 years from 2009 was conducted at the Sourô Sanou University Hospital Center (CHUSS) in Bobo-Dioulasso. HIV-1-positive patients with at least two viral load measurements and two CD4 T cell counts were included in this study. Excel 2019 and RStudio were used to analyze the data.

**Results:**

a total of 265 patients were included in this study. The mean age of the patients was 48 ± 8.98 years and women accounted for 77.7% of the study population. A considerable decrease in the number of patients with TCD4 lymphocytes below 200 cells/μl from year 2 of treatment and a progressive increase in those with TCD4 lymphocytes above 500 cells/μl were observed in the study. Regarding the evolution of viral load, an increase in the proportions of patients with an undetectable viral load and a decrease in those with a viral load greater than 1000 copies/ml were noticed in years 2, 5, 6, and 8 of the follow-up. However, a decrease in the proportions of patients with undetectable viral load and an increase in those with viral load above 1000 copies/ml were observed in the years 4, 7, and 10 of follow-up.

**Conclusion:**

this study highlighted the different trends of viral load and LTCD4 evolution over 10 years of antiretroviral treatment. It showed a good immunovirological response was shown at the beginning of antiretroviral therapy, and then, a poor evolution of these markers at certain periods during the follow-up of HIV-positive patients.

## Introduction

The progression of Human Immunodeficiency Virus (HIV) infection leads to a weakened immune system in HIV-infected patients and increases their vulnerability to opportunistic infections [1]. The consequences of HIV infection in terms of the cost of the quality of life and health care represent a public health problem [2].

Many countries have, therefore, developed control strategies such as access to effective antiretroviral (ARV) drugs for people living with HIV (PLWHIV). The ARV treatments aim to make the patient's plasma viral load (PVL) undetectable in order to ensure the restoration of immune responses and break the chain of transmission of the virus [3,4].

Unfortunately, therapeutic failures are still observed in patients living with HIV despite the considerable benefits of antiretroviral therapy the quality of life of people living with HIV (PLWHIV). This can result in poor outcomes of the evolution of immunological and virological markers. In Indonesia, Fibriani *et al*. reported 9.1% virological failure while in Nigeria Anude *et al*. 23.4% virological failure, 22.7% immunological failure and 33% immunovirological discordance among PLWHIV [5,6].

In Burkina Faso rate of 11.9% immunological failure, 8% virological failure and 9.5% immunovirological discordance were highlighted in one study [2]. The poor evolution of immunological and virological markers could constitute a challenge towards the 3x95´ objectives set by the World Health Organization (WHO).

Although some studies were carried out on the evolution of immunovirological markers in Burkina Faso, those on the long-term follow-up of people living with HIV remain scarce and not well-documented. This study aimed to describe the long-term evolution of immunological and virological parameters in patients undergoing treatments for HIV-1 at the Day Hospital of Bobo-Dioulasso in order.

## Methods

**Design of the study:** a retrospective descriptive and analytical study was conducted at the Sourô Sanou University Hospital Center (*CHUSS*) in Bobo-Dioulasso. It is one of the six (06) university hospital centers in Burkina Faso. Data was collected from November 1, to December 31, 2019 at the Day Hospital, which provides outpatient care for people living with HIV. The study protocol was approved by the head of the Infectious Diseases Department and the Immunology-Virology Laboratory. Informed consent forms were sought from all patients. The anonymity and confidentiality of the data collected were guaranteed and only the Evaluation and Operational Monitoring of ESTHER Programs (ESOPE) numbers were mentioned on the forms.

**Study population:** HIV-1 infected patients under antiretroviral treatment from 2009 to 2019 with at least two plasma viral loads and two CD4 counts were included in this study. Patients co-infected with HIV-1 and HIV-2, HIV-positive patients lost to follow-up, transferred or deceased HIV-positive patients were excluded in the study.

**Sample size:** a total of two hundred and sixty-five (265) subjects were randomly enrolled in this study without consideration to their age and gender.

**Variables:** the variables collected were the socio-demographic characteristics of the patients (age, sex, education level, place of residence and occupation) and biological data (CD4 count and plasma viral load).

**Data collection:** these social, demographic and biological data were retrieved from the computerized databases ESOPE (Evaluation and Operational Monitoring of ESTHER Programs) and the laboratory database.

**Laboratory analyses:** the LTCD-4 count in whole blood was determined using the BD FACSCount machine. Viral ribonucleic acid (RNA) was extracted from the plasma samples manually or semi-automatically using the Abbott m2000 Real Time extraction kit and the Viral NA Extraction® kit, respectively. The semi-automatic viral RNA extraction was performed on the Arrow® extractor using. Amplification and real-time detection of the viral RNA were performed on the m2000rt or CFx96 thermal cyclers depending on the availability of reagents.

**Data analysis:** Excel 2019 and R were used to analyze the data. Univariate regression analysis was carried out to identify the factors associated with low CD4 count and high viral load level, p<0.05 was considered as statistically significant.

## Results

A total of 265 patients were included in this study.

**Socio-demographic characteristics of the patients:** the socio-demographic characteristics of the study population are shown in Table 1. Women represented 77.7% of the participants. The average age of the patients was 48 ± 8.9 years with extremes of 29 and 75 years. The most common age group was 39 to 48 years with a frequency of 41.9%. The proportion of patients not attending school was 45.3%. Patients residing in Bobo-Dioulasso represented 92.1% of the study population. Married people were the most represented in marital status groups with 48.7% according to the marital status. Unemployed patients were the majority represented with 63.4%.

**Table 1:** sociodemographic characteristics of the study population

Parameters	Numbers	Percentage (%)	CI 95 %
**Sex**			
*Men	206	77.7	73.2 - 82.6
Women	59	22.3	16.6 - 26.0
**Age groups (years)**			
29-38	38	14.3	9.6 - 18.6
*39-48	111	41.9	36.1- 49.1
*49-58	81	30.6	24.4 - 35.8
59-68	31	11.7	7.9 - 1.8
69-78	4	1.5	0.0 - 3.0
**Educational level**			
*Not in school	120	45.3	38.5 - 52.1
Primary	80	30.2	25.3 - 35.6
Secondary	58	21.9	17.0 - 27.3
Superior	7	2.6	0.8 - 4.9
**Marital status**			
*Married	129	48.7	42.5 - 55.1
Single	60	22.6	18.1 - 28.5
Widowed	53	20.0	15.1 - 24.7
Divorced	17	6.4	3.6 - 9.6
Free union	6	2.3	0.8 - 4.5
Occupation			
*Unemployed	168	63.4	57.0 - 68.7
Employee	60	22.6	17.2 - 28.3
Trader	6	2.3	0.8 - 4.5
Pupil/student	7	2.6	1.1 - 4.9
Others	24	9.1	6.2 - 12.5

* : predominant group; variables are expressed in numbers (percentages); CI: confidence interval

**Figure 1:**
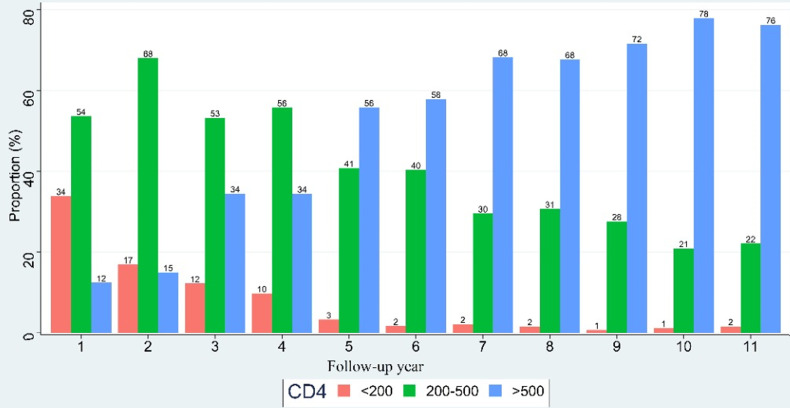
evolution of the proportions of patients per CD4 lymphocyte count/μl during the monitoring

**Evolution of the proportions of patients per CD4 lymphocyte count/μl during the monitoring** the proportions of patients per CD4 lymphocyte count/μl during follow-up period were indicated in the figure below (Figure 1). A considerable decrease in the number of patients with TCD4 lymphocytes below 200 cells/μl from year 2 of treatment and a progressive increase in those with TCD4 lymphocytes above 500 cells/μl were observed in the study.

**Figure 2:**
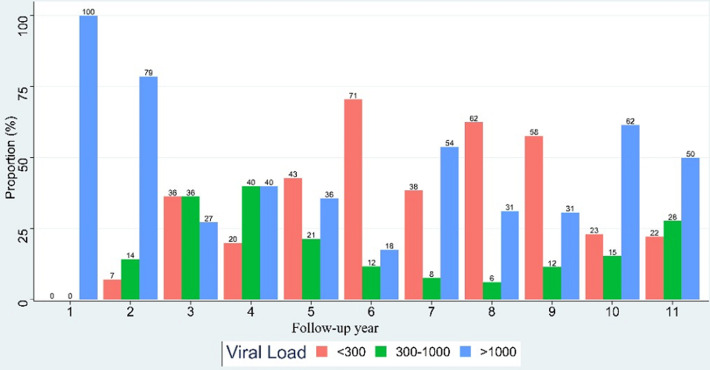
evolution of the proportions of patients by viral load band during the monitoring

**Evolution of the proportions of patients by viral load band during the monitoring:** regarding the evolution of viral load, an increase in the proportions of patients with an undetectable viral load and a decrease in those with a viral load greater than 1000 copies/ml were noticed in the year 2, 5, 6 and 8 of the follow-up. However, a decrease in the proportions of patients with undetectable viral load and an increase in those with viral load above 1000 copies/ml were observed in the year 4, 7 and 10 of follow-up (Figure 2).

**Factors associated with low TCD4 cell count and high viral load:** univariate analysis data of socio-demographic factors associated with TCD4 cell count and viral load level were indicated in the Table 2 and Table 3. None of the factors was associated with low CD4 cell count and high viral load in this study.

**Table 2:** factors associated with a low CD4 count

Characteristic	OR	95% CI	p-value
Age	1.01	0.98, 1.04	0.37
**Sex**			
Female	-	-	
Male	1.65	0.91, 2.96	0.1
**Profession**			
Housewife	-	-	
Trader and similarly	1.55	0.83, 2.88	0.16
Employee	1.38	0.56, 3.33	0.48
Other	0.75	0.29, 1.78	0.53
**Marital status**			
Single	-	-	
Monogamous married	1.1	0.57, 2.13	0.78
Polygamous married	1.04	0.42, 2.51	0.94
Widowed/divorced	1.08	0.53, 2.22	0.82
**Residence**			
Rural	-	-	
Urban	0.42	0.12, 1.36	0.15
**Education level**			
Not enrolled in school	-	-	
Primary	1.59	0.89, 2.88	0.12
Secondary and higher	1.74	0.93, 3.25	0.08

**Table 3:** factors associated with high viral load

Characteristic	OR	95% CI	p-value
Age	0.98	0.95, 1.02	0.29
**Sex**			
Female	-	-	
Male	1.47	0.71, 2.91	0.28
**Profession**			
Housewife	-	-	
Traders and similarly	0.93	0.41, 2.01	0.87
Employee	1.19	0.37, 3.27	0.74
Others	0.98	0.31, 2.62	0.97
**Marital status**			
Single	-	-	
Monogamous married	0.79	0.35, 1.83	0.57
Polygamous married	0.74	0.22, 2.23	0.61
Widowed/divorced	1.19	0.51, 2.80	0.69
**Residence**			
Rural	-	-	
Urban	1.17	0.30, 7.78	0.84
**Education level**			
Not enrolled in school	-	-	
Primary	0.81	0.38, 1.65	0.56
Secondary and higher	0.77	0.34, 1.66	0.52

## Discussion

The objective of the current study was to describe the long-term evolution of immunological and virological parameters in patients undergoing treatments for HIV-1 at the Day Hospital of Bobo-Dioulasso in Burkina Faso. A good immunovirological response was shown at the beginning of antiretroviral therapy, and then, a poor evolution of these markers at certain periods of during the follow-up of HIV-positive patients.

Our study population was predominantly consisted of female patients who accounted for 77.73%. A previous study conducted by Koné *et al*. in Côte d'Ivoire in 2019 reported a female predominance of 69% among people living with HIV with a prevalence [7]. Indeed, the vulnerability of women due to anatomical, physiological, socio-cultural and economic factors [2], the intervention of prevention of mother-to-child transmission which allows screening and care of women, poverty and prostitution could contribute to the feminization of HIV infection. In contrast, some authors such as Fibriani *et al*. mentioned a male predominance in a study conducted in Indonesia in 2013 [5]. This difference could lie in the intravenous drug use and homosexuality in northern countries. The mean age of the patients was 48 ± 8.98 years and the most dominant age group was 39-48 years making up 41.9% of the study population. Indeed, this age group is categorized as young and the subjects are the most sexually active with behavioural attitudes, which expose them to HIV infection [2].

The majority of the patients in the study were not in school and represented 43.5% of the study population followed by those with primary school level (30.2%). This high proportion of uneducated people in our study population could be due to the lack of information about HIV infection, which promotes risky behaviours among them. The study population was consisted of 63.4% unemployed patients and 13.2% people. These proportions of unemployment are greater than that of Ouédraogo *et al*. who reported 55.6% of unemployed people living with HIV from the same outpatient care setting [2]. We argue that several could explain this high proportion of unemployed PLWHIV. They include the high rate of unemployment rate in Burkina Faso and the public status of the HIV-positive patients healthcare setting. The latter often treats HIV-positive patients with limited income. In our study, 48.7% of married people represented the study population. Similar results were reported by Ouédraogo *et al*. in 2012 (54.2%) [2] and Diallo *et al*. (62.3%) [8]. In contrast, Rouveix *et al*. in their study on the analysis of motivations for the choice of ARVs prescribed to naive infected patients in 2016, reported that the category most affected was unmarried people (41%) [9].

During the follow-up period, we observed a decrease in the number of patients with CD4 < 200 cells/μl and an increase in the number of those with CD4 > 500 cells/μl from the second year of follow-up. Similar data were reported by Yapo *et al*. on the profile of routine monitoring biological parameters during follow-up of adolescents on long-term triple therapy in Abidjan in 2018. These results could indirectly reflect the effectiveness of antiretroviral therapy. Indeed, one of the objectives of ART is to restore the immune system of patients but only the viral load remains the indicator of its effectiveness [10].

Regarding the evolution of plasma viral load, our study reported an increase in the proportion of patients with undetectable viral load from the second year after initiation of ARV treatment. Our results are similar to that of Konaté on the rate of virological failures in adult patients undergoing ARV treatment at Sikasso Hospital in Mali in 2018 who reported a proportion of 74.4% of patients with an undetectable viral load at the second year of treatment [11]. Indeed, this increase of the proportion of patients with undetectable viral at the beginning of treatment could be due to the effectiveness of the initiated ARV treatment.

However, at years 4, 7 and 10, an increase in the proportion of patients with a viral load above 1000 copies/ml was observed. Our results are comparable to the data from Konaté in Mali 2018 who mentioned 63% of patients with a viral load above 1000 copies/ml followed up for more than 3 years [11]. These results could be explained by poor adherence to treatment which is the predictive factor of virological failures. Indeed, effective medical monitoring of people living with HIV requires regular visits to health care facilities. But this frequency becomes limited once the patients regain their complete health state, which translated to a “normal” life for them [12]. However, an increase in the number of patients with an undetectable viral load and a decrease in the number of those with a viral load above 1000 copies/ml in the years 5, 6 and 8 have been reported during follow-up. This could be due not only to possible changes in treatment regimens, but also to different retention strategies.

Given its retrospective nature, this study had some limitations including the lack of data on treatment lines and patient compliance. Non-adherence and changes in treatment lines could explain the variations in viral loads at certain periods of follow-up. This could lead to an underestimation of the factors associated with low CD4 count and high viral load level.

## Conclusion

This study highlighted the different trends of plasma viral load and LTCD4 evolution over 10 years of antiretroviral treatment. It showed a good immunovirological response was shown at the beginning of antiretroviral therapy, and then, a poor evolution of these markers at certain periods during the follow-up of HIV-positive patients. Although treatment is beneficial in the long term there is a poor immunological and virological response that can lead to a high rate of virological and immunological failure. For better patient management, it would be necessary to carry out a large-scale prospective study to better evaluate the factors that may be associated with these failures.

### What is known about this topic

- *The main objective of antiretroviral treatment is to suppress the plasma viral load, which allows better immune restoration*;
- *The monitoring of HIV-infected patients is mainly based on the measurement of the main immunological and virological markers, i.e. the TCD4 lymphocyte count and the plasma viral load*.

### What this study adds

- *At the Day Hospital of Bobo Dioulasso (Burkina Faso), the immunovirological characteristics of patients infected with HIV-1 and followed up on a long-term basis were documented*.
